# Transcriptome Analysis of the Oil-Rich Tea Plant, *Camellia oleifera*, Reveals Candidate Genes Related to Lipid Metabolism

**DOI:** 10.1371/journal.pone.0104150

**Published:** 2014-08-19

**Authors:** En-Hua Xia, Jian-Jun Jiang, Hui Huang, Li-Ping Zhang, Hai-Bin Zhang, Li-Zhi Gao

**Affiliations:** 1 Plant Germplasm and Genomics Center, Germplasm Bank of Wild Species, Kunming Institute of Botany, the Chinese Academy of Sciences, Kunming, China; 2 University of the Chinese Academy of Sciences, Beijing, China; ISA, Portugal

## Abstract

**Background:**

Rapidly driven by the need for developing sustainable sources of nutritionally important fatty acids and the rising concerns about environmental impacts after using fossil oil, oil-plants have received increasing awareness nowadays. As an important oil-rich plant in China, *Camellia oleifera* has played a vital role in providing nutritional applications, biofuel productions and chemical feedstocks. However, the lack of *C. oleifera* genome sequences and little genetic information have largely hampered the urgent needs for efficient utilization of the abundant germplasms towards modern breeding efforts of this woody oil-plant.

**Results:**

Here, using the 454 GS-FLX sequencing platform, we generated approximately 600,000 RNA-Seq reads from four tissues of *C. oleifera*. These reads were trimmed and assembled into 104,842 non-redundant putative transcripts with a total length of ∼38.9 Mb, representing more than 218-fold of all the *C. oleifera* sequences currently deposited in the GenBank (as of March 2014). Based on the BLAST similarity searches, nearly 42.6% transcripts could be annotated with known genes, conserved domains, or Gene Ontology (GO) terms. Comparisons with the cultivated tea tree, *C. sinensis*, identified 3,022 pairs of orthologs, of which 211 exhibited the evidence under positive selection. Pathway analysis detected the majority of genes potentially related to lipid metabolism. Evolutionary analysis of omega-6 fatty acid desaturase (*FAD2*) genes among 20 oil-plants unexpectedly suggests that a parallel evolution may occur between *C. oleifera* and *Olea oleifera*. Additionally, more than 2,300 simple sequence repeats (SSRs) and 20,200 single-nucleotide polymorphisms (SNPs) were detected in the *C. oleifera* transcriptome.

**Conclusions:**

The generated transcriptome represents a considerable increase in the number of sequences deposited in the public databases, providing an unprecedented opportunity to discover all related-genes associated with lipid metabolic pathway in *C. oleifera*. It will greatly enhance the generation of new varieties of *C. oleifera* with increased yields and high quality.

## Introduction

The tea-oil camellia (*Camellia oleifera*), a member of the family Theaceae, is well recognized as one of the world's four major woody oil tree together with oil palm, olive and coconut. It is an important and promoted woody oil plant in China, and has been widely utilized in many areas, such as food supplies, inks, lubricants, and cosmetics [1], [2]. The oil content of *C. oleifera* is up to 50%, usually serving as a high-quality cooking oil. More than 50% cooking oil in southern China, especially in Hunan Province, is tea-oil [3]. Tea oil contains several fatty acids, such as stearic, palmitic, oleic, linoleic and linolenic acids [4], [5]. Approximately ∼90% fatty acids of the *C. oleifera* are unsaturated fatty acid, comprising ∼83% (almost the highest among all natural oils) oleic acid and ∼7% linoleic acid [3], [5]. Besides, due to the similar oil composition between *C. oleifera* and olive, the oil of *C. oleifera* (tea-oil) has also been called “Oriental Olive Oil”. What's more, the tea oil can also reduce serum triglycerides and increase high-density lipoproteins (often regarded as good cholesterol) in humans [6]. Compared with olive and other corn oils, the oil of *C. oleifera* was also found to have a better stability against oxidation, leading to a suitable nutritional value [7]. Moreover, tea oil is still a good raw material for industrial uses and has been extensively utilized to manufacture soap, margarine, hair-oil, lubricants and rustproof oil. However, in contrast to its vital economic and strategic values, little information and genetic resources are available, which is mainly owing to the absence of genomic and/or transcriptomic resources for this non-model species.

Recent advances in high-throughput next-generation sequencing (NGS) technologies show a great potential for the large-scale production of genomic or transcriptomic data for a non-model species at reasonable costs [8]–[10]. Especially for the 454 pyrosequencing, of which the sequencing reads now approaching the length of traditional Sanger sequences, is ideal for the transcriptome sequencing for species that lacks a sequenced genome [11]. Besides, the latest versions of Newbler assembler from 454 can effectively assembly 454 RNA-Seq reads into putative transcripts, which can be better used for the subsequent gene discovery [12], [13], microarrays design [14] and high throughput SSRs or SNPs identification [15]–[17]. The SSRs and SNPs are often utilized as gene-based genetic markers and widely used for the generation of linkage maps and identification of quantitative trait loci (QTLs).

In this study, we sequenced and *de novo* assembled the transcriptome of *C. oleifera* using Roche/454 GS FLX massive parallel pyrosequencing platform for the first time. Our goals were to: 1) characterize the complete transcriptome of *C. oleifera*, and expand the genetic resources available for *Camellia* breeding programs; 2) identify the gene-based markers, including SSRs and SNPs, for future genetic analyses; 3) explore the dynamic evolution of orthologous genes between *C. oleifera* and *C. sinensis*, and assess the natural selection pressure assigning to genes during the *Camellia* evolutionary history; 4) discover the genes encoding enzymes that are involved in major metabolic pathways related to lipid biosynthesis and catabolism in *C. oleifera*, and further analyze the evolutionary process of oil quality determining gene (*FAD2*) among oil-plants. These datasets and results reported here will provide a public resource and information for future genetic and functional genomic studies in *C. oleifera*.

## Results and Discussion

### Transcriptome sequencing and *de novo* assembly

**Figure 1:**
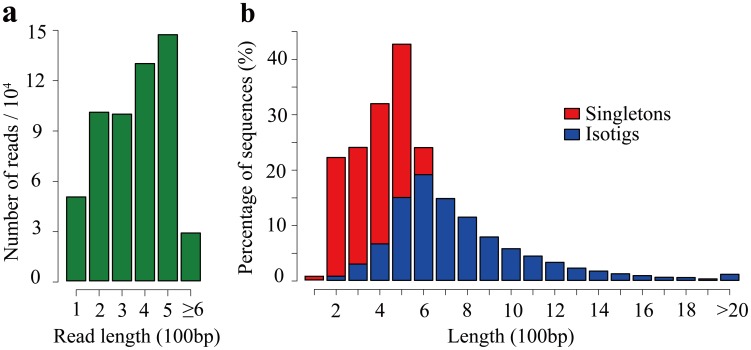
Overview of *C. oleifera* transcriptome sequencing and assembly. (**a**) Length distribution of 454 sequencing reads after filtering and trimming adapters. (**b**) Length distribution of the singletons and assembled isotigs.

To comprehensively generate the *C. oleifera* transcriptome, four cDNA libraries of tender shoots, young leaves, flower buds and flowers were normalized and sequenced using the 454 GS FLX platform. This resulted in 591,033 (∼186 Mb) raw reads with an average length of 315 bp. An overview of the sequencing and data pre-processing is shown in **Table 1**. After the removal of adaptors, primer sequences, poly-A tails as well as short and low quality sequences, a total of 554,198 (93.8%, ∼170 Mb) high-quality reads with an average length of 307 bp were retained and used for assembly. Size distribution for these trimmed, size-selected reads is shown in **Figure 1a**. For assembly, all trimmed and cleaned reads were mutually aligned and assembled using Newbler (version 2.8, Roche, IN, USA), yielding a set of 13,056 contiguous sequences (isotigs) longer than 100 bp, with an N50 of 771 bp. These results showed that more than half of the total assembly length of isotigs was >700 bp (**Table 2** **and** **Figure 1b**). Due to the fact that many genes in the transcriptome are expressed at levels low enough to hinder adequate sampling for 454 sequencing [18], reads that had no apparent overlapping with other reads in the database may contain useful gene information, which could not be obtained from isotigs. Considering this, the remaining 107,369 high-quality reads with length ≥100 bp were also retained and treated as singletons (i.e., reads not assembled into isotigs). Size distribution of these singletons is shown in **Figure 1b**. To combine the singletons and assembled isotigs and reduce the redundancy among them, CD-HIT [19] (version 4.6) was used to cluster these 120,425 sequences into 104,842 unigenes for further analyses. To our knowledge, so far, the number of sequences that are publicly available for *C. oleifera* is fewer than 500. Therefore, the transcriptome dataset reported here significantly expands the genetic resources available for *C. oleifera* in the public database, and can serve as a foundation for future investigation of gene expression, important pathways, molecular genetics and functional genomics in *C. oleifera*.

**Table 1:** Summary of the sequencing data of *C. oleifera*.

Library	Raw data			Clean data		
	No. of reads	Average length (bp)	Total length (bp)	No. of reads	Average length (bp)	Total length (bp)
**Tender shoots**	119,177	313.10	37,314,251	112,055	299.57	33,567,808
**Young leaves**	169,653	304.50	51,658,715	157,874	297.34	46,942,253
**Flower buds**	172,391	300.85	51,863,705	159,944	295.85	47,320,030
**Flowers**	129,812	349.95	45,427,523	124,325	342.00	42,519,448
**Total**	591,033	315.15	186,264,194	554,198	307.38	170,349,539

**Table 2:** Summary of *de novo* assembly and transcriptome annotation of *C. oleifera*.

	# Sequences
**Assembly**	
Total number of isotigs	13,056
Total isotigs length (bp)	9,442,537
Isotig N50 (bp)	771
Number of isotigs (length ≥500 bp)	9,786
Number of isotigs (length ≥1 kb)	2,095
Number of singletons	107,369
Total number of unigenes	104,842
Total unigenes length (bp)	38,944,469
GC content (%)	39.1

Annotation			
Public protein databases search		Isotigs	Singletons
BLASTx against NR	43,690	10,669	33,021
BLASTx against TAIR10	37,336	9,976	27,360
BLASTx against UniRef90	44,057	10,694	33,363
BLASTx against KOG	24,674	6,509	18,165
All annotated unigenes	44,707	10,739	33,968
Unigenes hit all four databases	23,385	6,428	16,957

Domain/functional annotation		Functional classification
Search against PFAM database	18,992	4,860 domains/families
Annotated with Gene Ontology (GO) terms	27,531	3 main categories
Assigned with Enzyme Commission (EC) numbers	9,993	323 pathways

### Functional annotation of the *C. oleifera* transcriptome

**Figure 2:**
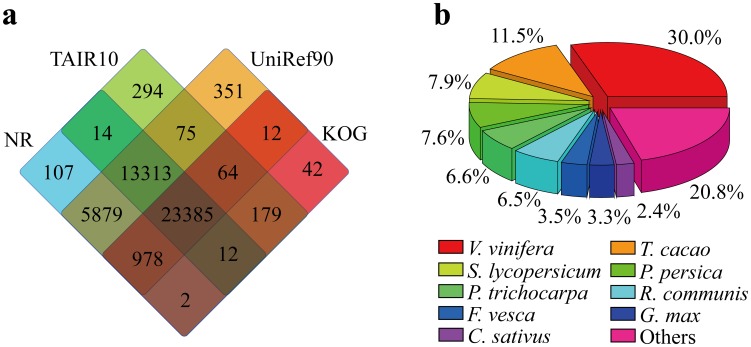
Characteristics of the homology search of unigenes against four public protein databases. (**a**) Venn diagram showing the BLAST searches of *C. oleifera* transcriptome against the four public protein databases. *De novo* reconstructed transcript sequences were used to BLAST against public databases including NCBI's NR, TAIR10, UniRef90 and KOG. The number of transcripts that have significant hits (E-value≤10^−5^) against the four databases is shown in each intersection of the Venn diagram. (**b**) Species distribution is shown as the percentage of the total homologous sequences (with an E-value≤10^−5^). We searched the NCBI NR database by using BLASTx and extracted the best hit of each sequence for analysis.

To comprehensively annotate the transcriptome of *C. oleifera*, several complementary approaches were adopted. First, the 104,842 non-redundant assembled sequences were aligned against four public protein databases, including National Center for Biotechnology Information (NCBI) non-redundant (NR) protein database, Arabidopsis Information Resource database (TAIR, version 10), UniRef90 and Clusters of Orthologous Groups (KOGs) protein database, using BLASTX algorithm [20]. With an E-value threshold of 10^−5^, a total of 10,739 isotigs (82.3% of total isotigs) and 33,968 singleton sequences (37.0% of total singletons) had the best BLAST matches with known proteins in at least one of the four databases, while 23,385 unigenes (22.3% of total unigenes; 6,428 isotigs plus 16,957 singletons) had significant BLAST matches to proteins in all of the four databases (**Table 2** **,** **Figure 2a** **and Table S1**). The top-hit species distribution of NR BLAST matches is shown in **Figure 2b**. Approximately 30% of the sequences had significant matches with genes from *Vitis vinifera*, followed by *Theobroma cacao* (11.5%), *Solanum lycopersicum* (7.9%), *Prunus persica* (7.6%), *Populus trichocarpa* (6.6%), *Ricinus communis* (6.5%), *Fragaria vesca* (3.5%), *Glycine max* (3.3%), and *Cucumis sativus* (2.4%).

Considering that the conserved domain information within a gene would be useful for interpreting the gene's function, we used the domain-based alignments to further detect the function of the *C. oleifera* transcriptome. To facilitate the domain annotation, we began by predicting the open reading frame (ORF) for each unigene, and then all the unigenes with detected ORFs were submitted to HMM search against the Pfam database [21] (version 27.0). In total, 18,992 matches were returned and categorized into 4,860 domains/families (**Table 2** **and Table S2**). The top ten most abundant domains/families are given in **Table 3**, which contained “Protein kinase domain”, “Protein tyrosine kinase”, “WD domain, G-beta repeat (WD40)”, “Cytochrome P450”, “RNA recognition motif”, “Reverse transcriptase, RNA-dependent DNA polymerase”, “UDP-glucoronosyl and UDP-glucosyl transferase”, “Mitochondrial carrier protein”, “ABC transporter”, and “PPR repeat family”.

**Table 3:** The ten most frequently occurred PFAM domains/families in the unigenes of *C. oleifera*.

Acc ID	Conserved domain/family	Sequences (n)
PF00069	Protein kinase domain	440
PF07714	Protein tyrosine kinase	220
PF00400	WD domain, G-beta repeat (WD40)	178
PF00067	Cytochrome P450	171
PF00076	RNA recognition motif (RRM_1)	151
PF07727	Reverse transcriptase, RNA-dependent DNA polymerase (RVT_2)	112
PF00201	UDP-glucoronosyl and UDP-glucosyl transferase	103
PF00153	Mitochondrial carrier protein	86
PF00005	ABC transporter	86
PF13041	PPR repeat family (PPR_2)	84

**Figure 3:**
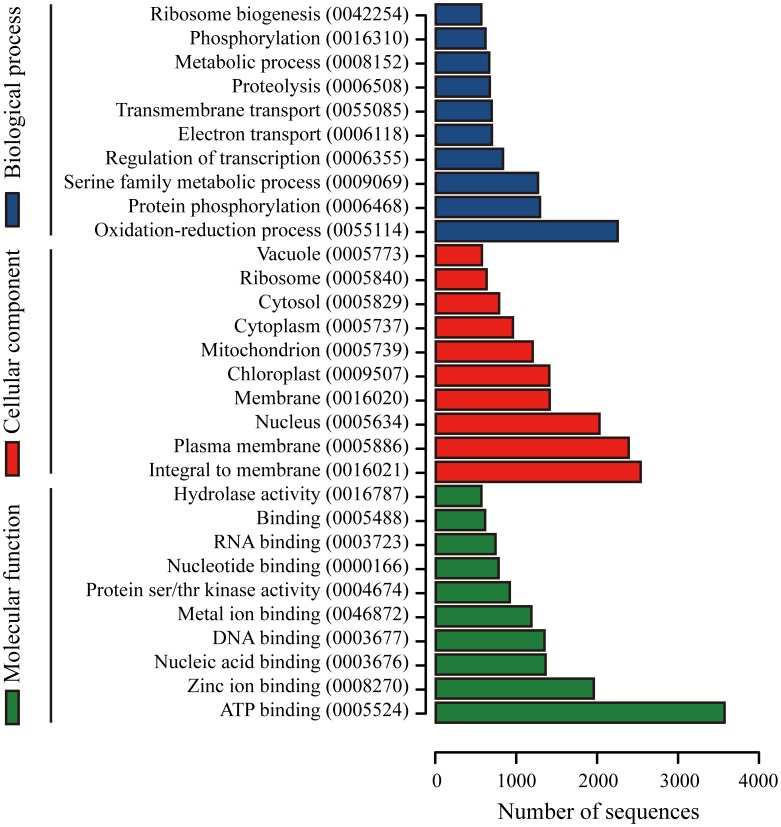
Histogram presentation of the most abundant Gene Ontology (GO) terms assigned to the *C. oleifera* transcriptome. The NR based Blast2GO results are summarized into three main GO categories: biological process (BP), cellular component (CC), and molecular function (MF). Only the top ten GO terms for each main function category are shown (blue: BP; red: CC; green: MF). The corresponding GO IDs are presented in parentheses. The x-axis indicates the number of genes assigned to the same GO term. One unigene may be matched to multiple GO terms.

Besides, we also used the Gene Ontology (GO) classification system to annotate the possible functions of the unigenes. Briefly, using the Blast2GO platform [22]–[24], sequences with a best match from NR database were further assigned with GO terms and Enzyme Commission (EC) numbers. Overall, 27,531 transcripts were assigned to 122,340 GO term annotations with an average number of 4.4 GO terms for each transcript (**Table 2** **and Table S3**). The distribution of ten most abundant GO terms for biological processes, molecular functions, and cellular components is presented in **Figure 3**. Additionally, of the 27,531 sequences annotated with GO terms, 9,993 sequences were assigned with EC numbers and involved in 323 different pathways when the Kyoto Encyclopedia of Genes and Genomes (KEGG) pathway mapping based on EC numbers for assignments was carried out (**Table 2**).

### Identification of SSRs, SNPs and Indels

**Figure 4:**
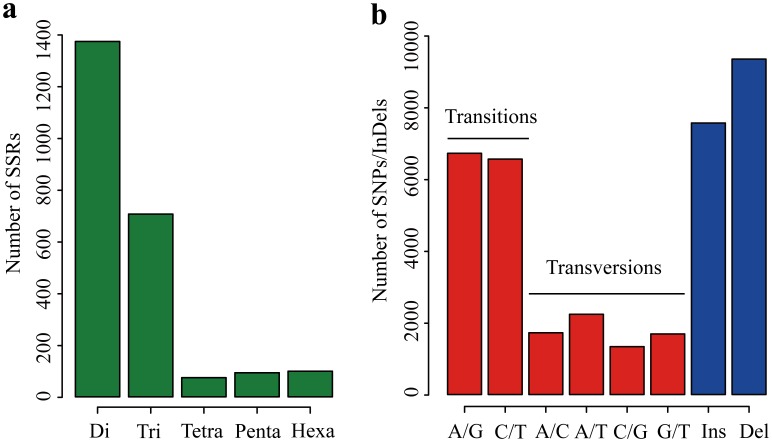
Distribution of simple sequence repeats (SSRs), single nucleotide polymorphisms (SNPs) and insertion/deletions (InDels) in *C. oleifera* isotigs. (**a**) Di-, tri-, tetra-, penta- and hexa-nucleotide repeats were analyzed. The x-axis shows the type of the SSRs, whereas y-axis shows total number of SSRs in different classes. (**b**) Frequencies of different SNPs/InDels. The x-axis indicates the substitution type of SNPs/InDels, while y-axis represents the number of SNPs/InDels for each substitution type.

Simple sequence repeats (SSRs) (also called microsatellites), consisting of repeated sequences of 2–6 bp in length, have been widely used in QTL analysis, evaluation of genetic variation and construction of genetic linkage maps [25]–[28]. Although *C. oleifera* is regarded as an important plant in China, few genetic markers are currently available for this species. The transcriptome data obtained by 454 sequencing provided an excellent source for mining and development of gene-based markers. Here, using a Perl script known as MicroSAtellite (MISA, http://pgrc.ipk-gatersleben.de/misa), we totally detected 2,345 SSRs from 13,056 assembled isotigs (**Table S4**). Of the 2,345 detected SSRs, di-nucleotide repeats were the most abundant type (1,373; 58.6%), followed by tri-nucleotides (706; 30.1%), hexa-nucleotides (99; 4.2%), penta-nucleotides (93; 4.0%) and tetra-nucleotide (74; 3.2%) (**Figure 4a**). Among the 1,373 di-nucleotide repeats, CT/AG (37.4%) was the most abundant motifs, followed by TC/GA (35.2%) and AT/AT (19.7%). GAA/TTC (10.5%) was the most common motif for tri-nucleotide repeats. Unlike other cereal species [29] like barley, maize, oats, rice, rye, wheat and sorghum, of which tri-nucleotide repeats are the most abundant class of microsatellite, the main SSR type of *C. oleifera* is di-nucleotide repeat. To the best of our knowledge, although there are much more microsatellites that were developed for the genus *Camellia* [30]–[32], relatively fewer are publicly available for *C. oleifera*, suggesting that the SSRs reported here will be particularly useful in the future genetic characterization and germplasm utilization.

Besides SSRs analysis, potential SNPs/Indels were also detected. In total, we identified 20,250 high-quality SNPs and 16,906 Indels from the *C. oleifera* transcriptome (**Table S5**). Among all the SNPs, transitions (65.6%) were more frequent than tranversions (34.4%) (**Figure 4b**). The number of SNPs detected per transcript was highly variable, ranging from 1 to 106; however, approximately 27.3% of the transcripts contained only one or two SNPs. Furthermore, of the predicted 20,250 SNPs, 15,364 (75.9%) were obtained from isotigs with NR annotation. These SNPs can be considered as priority candidates for marker development and should be very useful for future germplasm utilization and breeding programs for *C. oleifera*.

### Identification of orthologous genes and sequence divergence analysis between *C. oleifera* and *C. sinensis*

**Figure 5:**
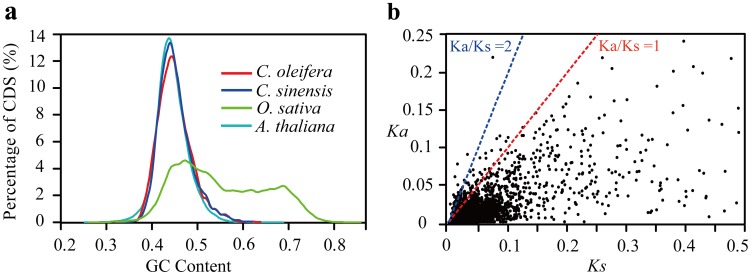
Characteristics of the 3,022 orthologous genes between *C. oleifera* and *C. sinensis*. (**a**) Distribution of GC content of CDS sequences among the *C. oleifera*, *C. sinensis*, *O. sativa* and *A. thaliana*. The CDS sequences of *O. sativa* (version 7.0) and *A. thaliana* (version 10) were downloaded from MSU Rice Genome Annotation Project (http://rice.plantbiology.msu.edu/) and TAIR (http://www.arabidopsis.org/), respectively. (**b**) Distribution of *Ka* and *Ks*. The mean *Ka*/*Ks* value is 0.39. The red line indicates the threshold of *Ka*/*Ks* = 1, whereas the blue line shows the more conservative threshold of *Ka*/*Ks* = 2. Analysis was performed using the method by Yang & Nielsen (2000).

Large-scale transcriptome sequencing of *C. sinensis* reported previously [33] provides a valuable source data for the comparative analysis of the evolution of *Camellia* orthologous genes. Using a reciprocal best hit (RBH) method with further relatively strict filters, we totally identified 3,022 putatively orthologous genes between *C. sinensis* and *C. oleifera* transcriptomes. Among the 3,022 orthologous genes, the average length was 645.6 bp with an average similarity of 95.74%. The overall GC content of *C. oleifera* in coding regions was 44.96%, consistent with *C. sinensis* (∼44.68%) and *A. thaliana* (∼44.11%) and much lower than *O. stativa* (∼52.96%) (**Figure 5a**). The 3,022 orthologous genes were also annotated with GO terms, and 1,717 orthologous were found to be involved in molecular functions, 1,571 orthologous were involved in biological processes, and 1,255 orthologous were involved in cellular components.

To understand the molecular evolution of orthologous genes between *C. oleifera* and *C. sinensis*, especially the genes undergoing purifying or positive selections, we calculated the synonymous (*Ks*) and non-synonymous (*Ka*) substitution rate for each orthologous gene pair. The mean value of *Ka/Ks* of these sequence pairs was 0.39. The *Ka/Ks* ratio (denoted as ω) is widely used to detect selective pressure acting on protein-coding sequences. *Ka/Ks*>1 indicates a sign of positive (adaptive) selection, *Ka/Ks*<1 shows a signature of negative (purifying) selection, and *Ka/Ks* = 1 means the neutral evolution. In our analysis, we found that the majority of sequences pairs (93%; 2,811/3,022) had a *Ka/Ks* ratio <1, suggesting most of the orthologous genes undergo purifying selection (**Figure 5b**). However, we also identified 211 sequences under accelerated evolution with ratios of *Ka/Ks*>1 and 38 sequences with ratios of *Ka/Ks*>2 (**Figure 5b** **and Table S6**). These sequences were found to be mainly involved in “ATP binding (GO: 0005524)”, “integral to membrane (GO: 0016021)” and “oxidation-reduction process (GO: 0055114)”. These fast evolving transcripts may be useful for identifying genes that perhaps strongly underwent positive selection during evolution process and might be responsible for speciation in the *Camellia* lineage.

### Metabolic pathways in *C. oleifera*

Composed with approximately 82∼84% unsaturated fatty acid [34] and a low content of saturated fat, the oil of *C. oleifera* has been wildly used in China for cooking oil, lubricants and cosmetics. Considering its high storage of lipids as potential new raw materials for biofuel production, we next focus on the pathways related to lipid metabolism, mainly including fatty acid and triacylglycerols (TAG) metabolic pathways.

#### Fatty acid biosynthesis and catabolism

The plant fatty acids possess highly edible and industrial values. However, free fatty acids are rarely found in nature but instead are often esterified to a phospholipid (membrane lipids) [35], [36] and glycerolipid (energy-storage) [37]. The *de novo* biosynthesis of fatty acids, up to a chain length of C16 or C18, from acetyl-CoA mainly involves two enzyme systems: acetyl-CoA carboxylase (ACC) and fatty acid synthase (FAS) complex. In higher plants, both of these two systems are located in the chloroplast and the majority of the component polypeptides are nuclear-encoded. Although the overall fatty acid biosynthesis pathways are well studied in eukaryotes [38], [39], much less are known in the *C. oleifera*.

**Figure 6:**
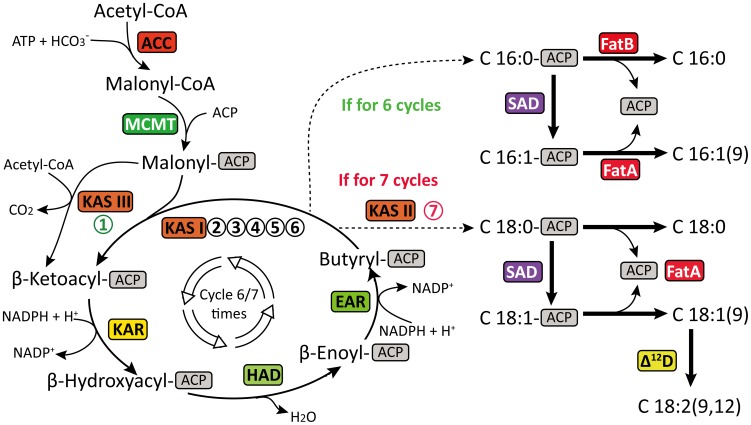
Core reactions of fatty acid biosynthesis reconstructed based on the *de novo* assembly and annotation of *C. oleifera* transcriptome. During fatty acid biosynthesis, two-carbon units are added for each cycle reaction, and the four-step cycle is repeated until the appropriate chain-length is reached. Finally, different types of fatty acids are synthesized. The identified enzymes are shown in boxes and abbreviated as below: ACC, acetyl-CoA carboxylase (EC: 6.4.1.2); MAT, malonyl-CoA ACP transacylase (EC: 2.3.1.39); KAS, beta-ketoacyl-ACP synthase (KAS I, EC: 2.3.1.41; KASII, EC: 2.3.1.179; KAS III, EC: 2.3.1.180); KAR, beta-ketoacyl-ACP reductase (EC: 1.1.1.100); HAD, beta-hydroxyacyl-ACP dehydrase (EC: 4.2.1.-); EAR, enoyl-ACP reductase (EC: 1.3.1.9); AAD, acyl-ACP desaturase (EC: 1.14.19.2); OAH, oleoyl-ACP hydrolase (EC: 3.1.2.14); FatA, Acyl-ACP thioesterase A (EC: 3.1.2.-); Δ^12^D, Δ^12^(ω^6^)-desaturase (EC: 1.4.19.6). The numbers-in-circles indicates the repeat time of the condensation reaction.

Based on the *de novo* assembly and functional annotation of the *C. oleifera* transcriptome, we have successfully identified multiply transcripts encoding the key enzymes involved in the fatty acid biosynthesis and catabolism pathway of *C. oleifera* (**Table 4** **and Table S7**). Fatty acid biosynthesis in *C. oleifera* was derived from the acetyl-CoA, which was initially catalyzed by the acetyl-CoA carboxylase (ACC, EC: 6.4.1.2) to form malonyl-CoA. Next, malonyl-CoA ACP transacylase (MCMT, EC: 2.3.1.39) catalyzed the transfer of acyl carrier protein (ACP) to the malonyl group, producing a malonyl-ACP, the primary substrate for the subsequent elongation. During the elongation process, four reactions were required with the addition of two carbons. First, the beta-ketoacyl-ACP synthase III (KAS III, EC: 2.3.1.180) catalyzed the initial condensation reaction, linked the acetyl group from acetyl-CoA to the malonyl-ACP, and yielded the beta-ketoacyl-ACP containing four carbons. Then, the ketoacyl-ACP was reduced by an NADPH-dependent beta-ketoacyl-ACP reductase (KAR, EC: 1.1.1.100) to generate the beta-hydroxyacyl-ACP. Next, 3-Hydroxyacyl-ACP dehydratase (HAD, EC: 4.2.1.-) removed a molecule of water from beta-hydroxyacyl-ACP to generate an enoyl-ACP, which was finally reduced by NADH to butyryl-ACP in a reaction catalyzed by enoyl-ACP reductase (EAR, EC: 1.3.1.9). The product of the first synthetic cycle, butyryl-ACP, was the substrate for further elongation rounds, each of which used one molecule of malonyl-ACP and released carbon dioxide. Notably, the condensations from C4 to C16 were carried out by beta-ketoactyl-ACP synthase I (KAS I, EC: 2.3.1.41), instead of the beta-ketoactyl-ACP III, although most enzymes used for the further elongation were the same as utilized for generating butyryl-ACP from acetyl-CoA and malonyl-ACP (**Figure 6**). While the reaction from C16 to C18 was catalyzed by beta-ketoactyl-ACP synthase II (KAS II, EC: 2.3.1.179).

**Table 4:** Enzymes involved in fatty acid biosynthesis and catabolism identified by the annotation of the *C. oleifera* transcriptome.

Enzyme	Symbol	EC Number	Number of unigenes
**Fatty acid biosynthesis**			
Biotin carboxylase	BC	6.3.4.14	1
Biotin carboxyl carrier protein	BCCP	6.4.1.2	8
Acetyl-CoA carboxylase	ACC	6.4.1.2	17
malonyl-CoA-ACP transacylase	MCMT	2.3.1.39	3
Beta-ketoacyl-ACP synthase I	KAS I	2.3.1.41	7
Beta-ketoacyl-ACP synthase II	KAS II	2.3.1.179	3
Beta-ketoacyl-ACP synthase III	KAS III	2.3.1.180	7
Beta-ketoacyl-ACP reductase	KAR	1.1.1.100	8
3R-hydroxyacyl-ACP dehydrase	HAD	4.2.1.17; 4.2.1.-	2
Enoyl-ACP reductase (NADH)	EAR	1.3.1.9	4
Acyl-ACP thioesterase A	FatA	3.1.2.14	4
Acyl-ACP thioesterase B	FatB	3.1.2.14	5
**Fatty acid desaturation**			
Stearoyl-ACP Desaturase	SAD	1.14.19.2	10
Δ^12^ (ω6)-Desaturase	Δ^12^ D	1.14.19.6	3
**Fatty acid catabolism**			
Long-chain acyl-CoA synthetase	LACS	6.2.1.3	6
Acyl-CoA oxidase	ACX	1.3.3.6	12
Enoyl-CoA hydratase	ECH	4.2.1.17	6
3s-hydroxyacyl-CoA dehydrogenase	HACDH	1.1.1.35	6
Ketoacyl-CoA Thiolase	KAT	2.3.1.16	12
Acyl-CoA Thioesterase	ACT	3.1.2.2	4
Enoyl-CoA isomerase	Isom	5.3.3.8	4
Dienoyl CoA Isomerase	DCI	5.3.3.8; 4.2.1.17	1
Dienoyl-CoA Reductase	Red	1.3.1.34	9
Trans-2-enoyl-CoA reductase	ECR	1.3.1.38	4
Enoyl-CoA Hydratase 2	ECH2	4.2.1.17	5

For the synthesis of unsaturated fatty acids in plastid, a double bond was introduced to the acyl group esterified to ACP via acyl-ACP desaturase (AAD, EC: 1.14.19.2). The elongation of fatty acids was terminated when the acyl group was removed from the ACP by acyl-ACP thioesterase enzyme, oleoyl-ACP hydrolase (OAH, EC: 3.1.2.14), or when acyl-ACP released the free fatty acid. The final fatty acid composition was determined by the activities of enzymes that used these acyl-ACPs at the termination phase of fatty acid synthesis. We also identified desaturation enzymes Δ^12^(ω^6^)-desaturase (Δ^12^D/FAD2, EC: 1.4.19.6), which desaturated oleic acid (C18:1n-9) to generate linoleic acid (C18:2n-6). The above description showed the pathway responsible for the formation and conversion of fatty acid in *C. oleifera* and was depicted in **Figure 6**. We failed to identify any genes encoding enzymes involving further desaturation and elongation of linoleic acid to form longer chain polyunsaturated fatty acid in our annotated datasets. This is probably related to the certain components of fatty acids in *C. oleifera*, or the lack of transcriptome sequences from libraries of seed tissues in some case. Besides synthesis, we also identified all the enzymes involving fatty acid catabolism (namely, beta-oxidation pathway) in *C. oleifera* (**Table 4** **and Table S7**). The beta-oxidation pathway in *C. oleifera* involved four enzymes, including acyl-CoA oxidase (ACX, EC: 1.3.3.6), enoyl-CoA hydratase (ECH, EC: 4.2.1.17), 3-hydroxyacyl-CoA dehydrogenase (HACDH, EC: 1.1.1.35), and ketoacyl-CoA thiolase (KAT, EC: 2.3.1.16).

Together, the *C. oleifera* transcriptome presented here covers most of the enzymes required for the biosynthesis, elongation and metabolism of fatty acids (**Table 4** **and Table S7**). These identified enzymes contribute to the biochemical and molecular information needed for metabolic engineering of fatty acid synthesis. For example, linoleic acid, or omega-6 fatty acid, is an essential fatty acid for people's health. With the lack of Δ^12^(ω^6^)-desaturase (Δ^12^D, EC: 1.4.19.6), they cannot be synthesize by human body and have to be obtained from the diet. Further genetic engineering with these enzymes will contribute to oil composition, satisfy people's demands for oil and improve our life.

#### TAG biosynthesis and catabolism

**Figure 7:**
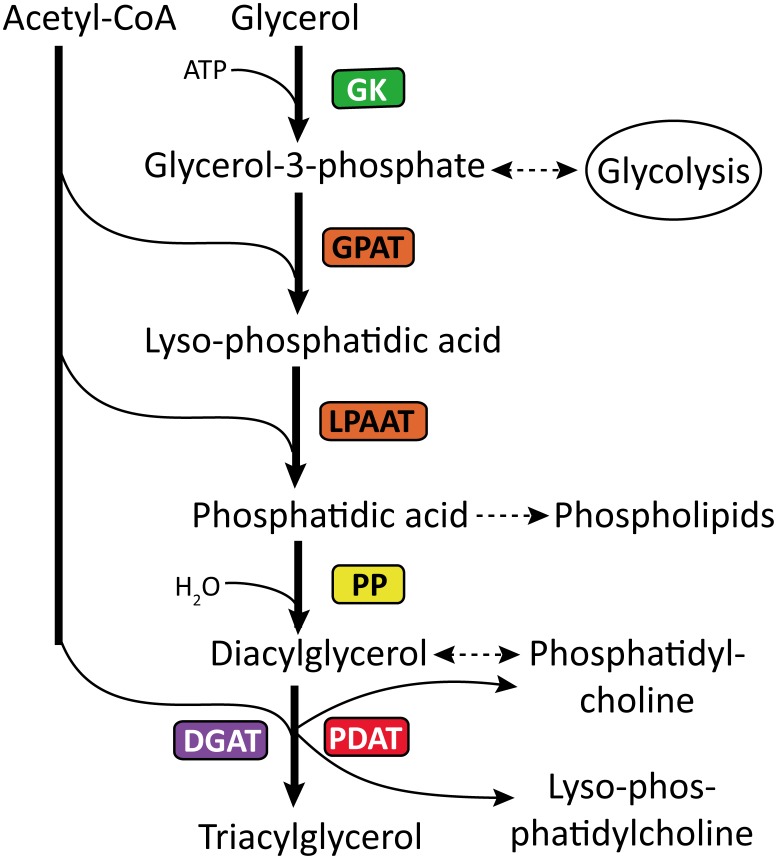
Triacylglycerol (TAG) biosynthesis pathway reconstructed based on the *de novo* assembly and annotation of *C. oleifera* transcriptome. Identified enzymes are shown in boxes, including: GK, glycerol kinase (EC: 2.7.1.30); GPAT, glycerol-3-phosphate O-acyltransferase (EC: 2.3.1.15); AGPAT, 1-acyl-sn-glycerol-3-phosphate O-acyltransferase (EC: 2.3.1.51); PP, phosphatidate phosphatase (EC: 3.1.3.4); DGAT, diacylglycerol O-acyltransferase (EC: 2.3.1.20); and PDAT, phopholipid∶ diacyglycerol acyltransferase (EC: 2.3.1.158). The dashed arrows denote reaction(s) in which the enzymes are not shown.

Nearly all eukaryotic organisms and even a few prokaryotes have the ability to synthesize triacylglycerols [40], leaving alone the important oil-plant, *C. oleifera*. Despite the global process for TAG biosynthesis in *C. oleifera* has been known, however, merely fewer genes were cloned. Here, according to the functional annotation of *C. oleifera* transcriptome, transcripts encoding most of the enzymes involving in the TAG biosynthesis pathway were also identified. **Table 5** **and Table S7** lists all the enzymes obtained above, and **Figure 7** shows the TAG pathway reconstructed based on the determined transcripts. The most important route to triacylglycerol biosynthesis is the glycerol-3-phosphate (G-3-P) pathway. Within this pathway, the glycerol-3-phosphate was first produced by either the catabolism of glucose (glycolysis) or the action of the enzyme glycerol kinase (GK, EC: 2.7.1.30) on free glycerol, and subsequently catalyzed by a glycerol-3-phosphate acyltransferase (GPAT, EC: 2.3.1.15) at the position *sn*-1 to form lyso-phosphatidic acid. The lyso-phosphatidic acid was continually acylated by an acylglycero-3-phosphate acyltransferase (LPAAT, EC: 2.3.1.51) at the position *sn*-2 to form phosphatidic acid (PA). And then the phosphate group of the PA was removed by an enzyme called phosphatidic acid phosphohydrolase (PP) to generate diacylglycerols (DAG). After the synthesis of DAG, the formation of TAG could occur in two ways (**Figure 7**). In one pathway, diacylglycerol acyltransferase (DGAT) transferred an acyl group from acetyl-CoA to *sn*-3 of DAG to form TAG. Another pathway involved a phospholipid∶diacylglycerol acyltransferase (PDAT) that utilized phospholipid as the acyldonor in TAG formation. It is worth mentioning that the last step of TAG pathway was an important determinant of cellular oil content and quality. These genes can be as priority candidates for the further cloning.

**Table 5:** Enzymes involved in TAG biosynthesis and catabolism identified by the annotation of the *C. oleifera* transcriptome.

Enzyme	Symbol	EC Number	Number of unigenes
**TAG biosynthesis**			
Glycerol kinase	GK	2.7.1.30	6
Glycerol-3-phosphate O-acyltransferase	GPAT	2.3.1.15	1
1-Acyl-sn-glycerol-3-phosphate O-acyltransferase	LPAAT2	2.3.1.51	5
	LPAAT4	2.3.1.51	3
	LPAAT5	2.3.1.51	3
Phosphatidate phosphatase	PP	3.1.3.4	5
Diacylglycerol O-acyltransferase	DGAT1	2.3.1.20	2
	DGAT2	2.3.1.20	1
	DGAT3	2.3.1.20	1
Phospholipid∶diacyglycerol acyltransferase	PDAT1	2.3.1.158	13
Monoacylglycerol Acyltransferase	MAGAT	2.3.1.22	1
Diacylglycerol Cholinephosphotransferase	DAG-CPT	2.7.8.2	1
Phosphatidylcholine∶diacylglycerol cholinephosphotransferase	PDCT	2.7.8.*	1
1-Acylglycerol-3-Phosphocholine Acyltransferase	LPCAT1	2.3.1.23	3
Choline Kinase	CK1	2.7.1.32	4
Choline-Phosphate Cytidylyltransferase	CCT1	2.7.7.15	4
**TAG catabolism**			
Triacylglycerol lipase	TAGL	3.1.1.3	18
Monoacylglycerol Lipase (MAGL)	MAGL	3.1.1.23	20

### Evolution of the omega-6 fatty acid desaturase 2 (*FAD2*) genes

**Figure 8:**
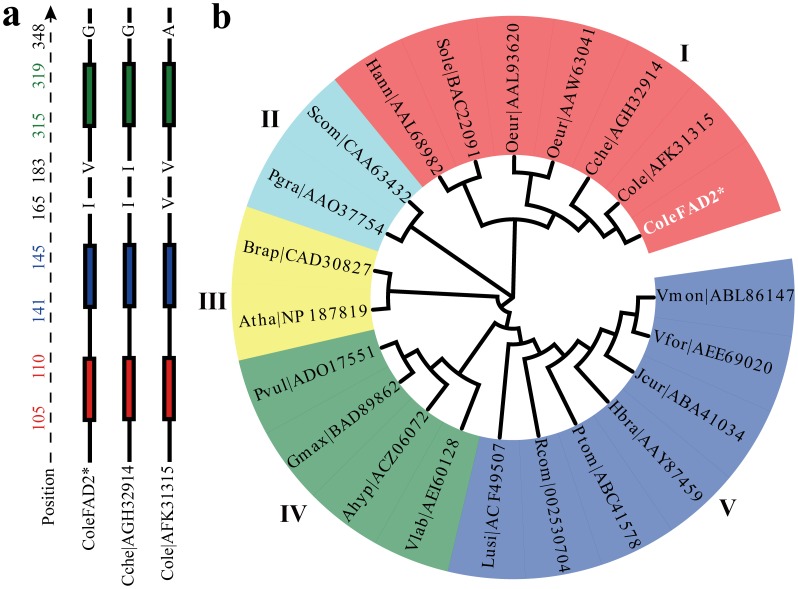
Phylogenetic analyses of the *FAD2* genes among 20 oil-plants. (**a**) The alignment of Cole|AFK31315 (*C. oleifera*, AFK31315), Cche|AGH32914 (*C. chekiangoleosa*, AGH32914) and ColeFAD2 (ColeIsotig4522:451–1599) amino acid sequences. The solid black lines indicate conserved amino acids. The filled boxes represent three H-boxes, including HECGH (red box), HRRHH (blue box), and HVAHH (green box). The position (left) is based on *FAD2* gene in *C. chekiangoleosa* (AGH32914). The three inconsistent amino acids were plotted in uppercase letters (black). Multiple sequence alignment was performed using ClustalW [58], [59] package. (**b**) The amino acid sequences were used for phylogenetic tree analysis. The asterisk indicates the *FAD2* gene (ColeFAD2) detected in the assembled *C. oleifera* transcriptome (ColeIsotig4522:451–1599). I–V represent the five groups of all the 20 oil-plants classified by the sequence similarity. The GenBank accession numbers and the full species names of the genes used here are: Scom|CAA63432 (*Solanum commersonii*, CAA63432); Atha|NP_187819 (*Arabidopsis thaliana*, NP_187819); Hann|AAL68982 (*Helianthus annuus*, AAL68982); Brap|CAD30827 (*Brassica rapa*, CAD30827); Sole|BAC22091 (*Spinacia oleracea*, BAC22091); Oeur|AAL93620 (*Olea europaea*, AAL93620); Pgra|AAO37754 (*Punica granatum*, AAO37754); Oeur|AAW63041 (*Olea europaea*, AAW63041); Gmax|BAD89862 (*Glycine max*, BAD89862); Hbra|AAY87459 (*Hevea brasiliensis*, AAY87459); Jcur|ABA41034 (*Jatropha curcas*, ABA41034); Ptom|ABC41578 (*Populus tomentosa*, ABC41578); Vmon|ABL86147 (*Vernicia Montana*, ABL86147); Lusi|ACF49507 (*Linum usitatissimum*, ACF49507); Rcom|002530704 (*Ricinus communis*, XP_002530704); Ahyp|ACZ06072 (*Arachis hypogaea*, ACZ06072); Pvul|ADO17551 (*Phaseolus vulgaris*, ADO17551); Vfor|AEE69020 (*Vernicia fordii*, AEE69020); Vlab|AEI60128 (*Vitis labrusca*, AEI60128); Cole|AFK31315 (*C. oleifera*, AFK31315); Cche|AGH32914 (*C. chekiangoleosa*, AGH32914).

The annotation of the *C. oleifera* transcriptome also identified a gene of *FAD2*. The coding sequence (CDS) of this putative *FAD2* gene was located in 451–1599 bp of ColeIsotig4522, so-called as ColeFAD2. Besides, two *FAD2*-like fragments (ColeSingleton23567 and ColeIsotig8486) were also detected in this transcriptome with an average length of 428 bp. Alignment of the deduced polypeptide sequence of the ColeFAD2 together with other two previously cloned *FAD2* genes (AFK31315 and AGH32914) displayed extremely high similarity (99.5% and 99.7%, respectively), suggesting that it indeed encodes microsomal oleate desaturase. Despite such high similarities among them, there still existed three inconsistent amino acids (**Figure 8a**). The amino acid Ile (I) at the position 165 of AFK31315 has been converted to a Val (V). In contrast to AGH32914, the amino acid Val (V) at the position 183 has been changed to an Ile (I). And the last inconsistent amino acid appeared in AFK31315 at the position of 348, where the amino acid Gly (G) has been converted to an Ala (A). Similar to the homologous *FAD2* genes in other plants, the putative polypeptides of ColeFAD2 also contains three highly conserved histidine-rich motifs (namely, H-boxes; HXXXH, HXXHH, and HXXHH) with conserved spaces between them, which has been shown essential for desaturase activity [41] and responsible for the formation of the diiron-oxygen complex used in biochemical catalysis [42] (**Figure 8a** **and Figure S1**).

To investigate phylogenetic relationships of the orthologous *FAD2* genes, the deduced amino acid sequence of the identified *FAD2* gene (ColeFAD2) was aligned with 21 orthologous *FAD2* sequences from 20 oil-plants. Based on the sequence alignments, an unrooted neighbor-joining (NJ) tree was constructed using MEGA software [43] (version 5.05). As illustrated in **Figure 8b**, these 22 *FAD2* genes were mainly classified into five groups. Among these five groups, the three *Camellia FAD2* genes and two *Olive FAD2* genes, together with each of *Spinacia oleracea* and *Helianthus annuus FAD2* gene, were assigned to Group I, suggesting that they may share analogous functions leading to the similar components of fatty acid, which is regarded as a key factor determining the quality of edible oil. Furthermore, when excluding the two *Camellia* species from the un-rooted tree, *C. oleifera* was surprisingly found to be quite close to the *Olea europaea* but much far from *Vernicia montana*. This result indicates that parallel evolution may occur between *C. oleifera* and *Olive FAD2* genes, resulting in the creation of analogous structures that have a similar form or function for oil production.

### Gene validation and expression analysis

**Figure 9:**
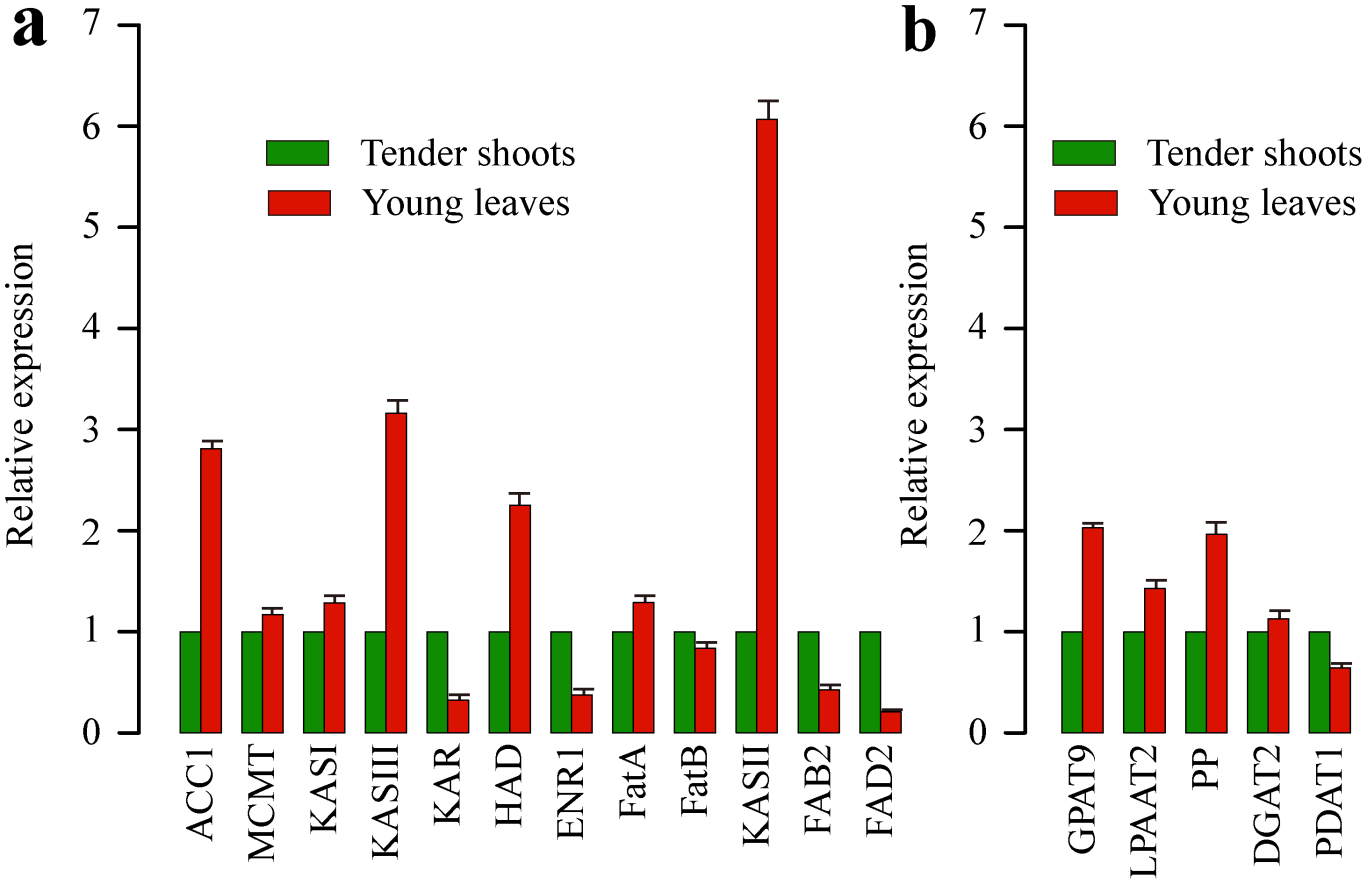
Quantitative RT-PCR validations of the 17 candidate lipid-related genes in the *C. oleifera* transcriptome. 17 candidate unigenes involved in lipid metabolism including (**a**) fatty acid and (**b**) TAG pathways were selected for the quantitative RT-PCR analysis. Standard error of the mean for three biological replicates (nested with three technical replicates) is represented by the error bars. Results represent the mean (± SD) of the three experiments. The translation elongation factor 1-alpha (TEF) gene was chosen as an internal standard.

To experimentally confirm that the unigenes obtained from the transcriptome sequencing and assembly were indeed expressed, 17 key unigenes involved in the biosynthesis and metabolism of tea oil were chosen for RT-PCR and qRT-PCR analyses (**Figure 9**). The detailed information of the selected unigene ID and designed primer pairs in this study were given in **Table S8**.

In the RT-PCR analyses, single bands with the expected sizes were amplified for all 17 unigenes sampled (**Table S8**), suggesting that the assembled unigenes were reliable and subsequent experiments of gene expression were feasible. In the qRT-PCR analysis, relative expression levels of the selected unigenes were compared between two different tissues. In the fatty acid pathway, unigenes of *ACC1*, *KAS III*, *HAD* and *KAS II* were expressed much higher in young leaves than in tender shoots (**Figure 9a**), and the highest level of gene expression was observed in *KAS II*. Expression levels of *MCMT*, *KAS I* and *FatA* in young leaves were slightly higher than those in tender shoots. In comparison, expression levels of *KAR*, *ENR*, *FatB*, *FAB2* and *FAD2* were low in young leaves but high in tender shoots. For TAG pathway, five selected unigenes had the similar expression pattern except *PDAT1* (**Figure 9b**). The overall expression levels of *GPAT9*, *LPAAT2*, *PP* and *DGAT2* were higher in young leaves than in tender shoots, while *PDAT1* was expressed lower in young leaves but higher in tender shoots. The results of qRT-PCR expression analysis showed that the selected lipid metabolism related unigenes were indeed expressed in the assembled *C. oleifera* transcriptome and exhibited different expression patterns during the developmental stage of leaves.

## Conclusions

Here we sequenced and annotated the transcriptome of *C. oleifera*. A total of 2,345 SSRs, 20,250 SNPs and 16,906 Indels were detected and can be considered as candidate genetic markers for the future studies. To examine the dynamic evolution of othologous genes between *C. sinensis* and *C. oleifera*, we identified 3,022 pairs of orthogous genes, of which 211 were under positive selection with *Ks/Ks* ratio >1, suggesting that they may probably play a vital role in the evolutionary process of *Camellia* species. Given the fact that *C. oleifera* is an important oil-plant in China, thus, in this paper, we mainly focused on the pathways related to lipid metabolism and have successfully identified transcripts associated with fatty acid metabolism, oil accumulation and breakdown in *C. oleifera*. Moreover, given the economic significance of *C. oleifera* in the future, it requires further agronomic improvement. To achieve this goal, the genetic and functional genomic resources, especially those genes involved in oil synthesis, accumulation and breakdown as described in this paper, are becoming more important than before. For example, using the genetic engineering technology with genes related to oil synthesis pathway, *C. oleifera* can be genetically modified to produce transgenic plant with improved oil content and/or composition. Furthermore, due to the importance of gene expression regulation, the entire oil synthesis pathway in *C. oleifera* can also be engineered in the level of gene expression to increase expression of enzymes responsible for the synthesis of the oleic acid as well as linoleic acids and to decrease expression of enzymes responsible for the breakdown of such compounds. Overall, the *C. oleifera* transcriptome reported here provides an invaluable new resource for the further genetic engineering and molecular cloning as well as the future functional genomic researches.

## Materials and Methods

### Plant materials collection, cDNA library construction and 454 sequencing

Four tissues of *C. oleifera*, including tender shoots, young leaves, flower buds and flowers, were harvested in 2010 from East Park of Kunming Botanic Garden, Yunnan Province, China. All necessary permits were obtained from Wei-bang Sun, who is the director of the Kunming Botanical Garden, the Chinese Academy of Sciences. All samples were flash frozen in liquid nitrogen and stored at −80°C for RNA extraction. Total RNA was extracted by a modified CTAB method. The quality and quantity of total RNA were analyzed using agarose gel electrophoresis and Agilent 2100 Bioanalyzer RNA chip (Agilent Technologies, CA). cDNA library construction and normalization were performed as described previously [44]. All libraries were combined into a single pool and sequenced using the 454 GS-FLX platform (Roche, IN, USA).

### Sequence data processing and *de novo* assembly

The raw reads obtained were first pre-screened to remove adaptors, poly-A tails and contaminants using Seqclean (http://compbio.dfci.harvard.edu/tgi/software/). Low-quality (phred score <20) and short (length <60 bp) reads were trimmed using SolexaQA package [45] (-h 20; -l 60). The trimmed and size-selected reads were then *de novo* assembled using Newbler software (version 2.8), which performs best for restoring full-length transcripts [44]. Assembled isotigs and singletons were merged and the redundancy among them was removed by CD-HIT (version 4.6) [19] with the similarity threshold of 0.9. The combined and redundancy-removed unigenes were used for the later analysis.

### Data deposit

The dataset of 454 sequencing reads were deposited in the NCBI Short Read Archive (SRA) database with the accession number: SRR1472854, SRR1472847, SRR1472843 and SRR1472842. The assembled sequences were available at the NCBI Transcriptome Shotgun Assembly (TSA) database that can be accessed under the accession number GBHI00000000. The version described in this paper is the first version, GBHI01000000.

### Unigene functional annotation and pathway assignments

All distinct unigenes (>100 bp) were compared against the NR, Uniref90, TAIR10 and KOG protein databases using the Blastx program with a threshold E-value of 10^−5^. Gene names were assigned to each unigene according to known genes from the best Blastx hit. The ORF of the unigenes was predicted using ESTscan [46] (version 3.0.3) with a pre-built model for *A. thaliana* that is distributed with the package, and then searched against PFAM [21] (version 27.0) databases for domain/family annotation using HMMER [47] (version 3.0) with the default parameters. To annotate the unigenes with GO terms, the best Blastx hit from NR database for each transcript was submitted to BLAST2GO platform [22]–[24], and GO terms for each unigene were retrieved based on the relationship between gene names and GO terms. EC number was assigned and parsed based on the BLAST2GO results. To determine metabolic pathways, Kyoto Encyclopedia of Genes and Genomes (KEGG) mapping was used. The sequences with corresponding ECs obtained from Blast2GO were mapped to the KEGG metabolic pathway database. To further enrich the pathway annotation and to identify the BRITE functional hierarchies, assembled sequences were also submitted to the online KEGG Automatic Annotation Server [48] (KAAS; http://www.genome.jp/kegg/kaas/) with bi-directional best hit (BBH) method. The output includes KO assignments and KEGG pathways that are involved with the KO assignments.

### Identification of SSRs, SNPs and InDels

Five types of SSRs from di-nucleotides to hexa-nucleotides were identified using the MISA Perl script (http://pgrc.ipk-gatersleben.de/misa/). The minimum repeat unit size for di- nucleotides was set at six, at five for tri- to tetra-nucleotides, and at four for penta- to hexa-nucleotides. Putative SNPs and Indels were detected using ssahaSNP software [49] with the default parameters.

### Identification of orthologous genes between *C. oleifera* and *C. sinensis*

To identify genes that are putatively orthologous between *C. oleifera* and *C. sinensis*, a reciprocal best hit (RBH) method based on the Blastn program was used. Briefly, each sequence from *C. oleifera* was initially searched against all sequences from *C. sinensis* using Blastn, and conversely each sequence of *C. sinensis* was searched against all sequences from *C. oleifera*. Pairs of sequences that were the best hits each other and sequences longer than 300 bp were regarded as putative orthologs. Due to the limitation of RBH method [50], potential paralogs may not be completely excluded from the ortholog dataset. To remove the possible paralogs, all pairs of sequences were first searched against all plant protein sequences available in GeneBank and only pairs of sequences unambiguously mapped to the same protein with an E-value<1×10^−15^ were selected as orthologous genes. Similar method and criteria have been used in previous studies [51]–[53].

### Estimation of synonymous and non-synonymous substitution rates between orthlogous

To calculate the synonymous (*Ks*) and non-synonymous (*Ka*) substitution rate for each orthologous gene pair, each member of a pair of sequences was first searched against all plant protein sequences available in GenBank using Blastx with a significant E-value threshold of 10^−15^, and the CDS of each orthologous genes was determined based on the best alignment regions. After removing short (<300 bp) and unexpected stop codon(s) containing CDS, the rate of synonymous and non- synonymous substitution was estimated using the maximum likelihood method implemented in codeml program [54] under the F3x4 model [55].

### Multiple sequence alignment and phylogenetic analysis of *FAD2* genes

Amino acid sequences of the 21 *FAD2* genes from 20 oil-plants were downloaded from NCBI GenBank database. The accession numbers and the full species names for these genes were: Scom|CAA63432 (*Solanum commersonii*, CAA63432); Atha|NP_187819 (*Arabidopsis thaliana*, NP_187819); Hann|AAL68982 (*Helianthus annuus*, AAL68982); Brap|CAD30827 (*Brassica rapa*, CAD30827); Sole|BAC22091 (*Spinacia oleracea*, BAC22091); Oeur|AAL93620 (*Olea europaea*, AAL93620); Pgra|AAO37754 (*Punica granatum*, AAO37754); Oeur|AAW63041 (*Olea europaea*, AAW63041); Gmax|BAD89862 (*Glycine max*, BAD89862); Hbra|AAY87459 (*Hevea brasiliensis*, AAY87459); Jcur|ABA41034 (*Jatropha curcas*, ABA41034); Ptom|ABC41578 (*Populus tomentosa*, ABC41578); Vmon|ABL86147 (*Vernicia Montana*, ABL86147); Lusi|ACF49507 (*Linum usitatissimum*, ACF49507); Rcom|002530704 (*Ricinus communis*, XP_002530704); Ahyp|ACZ06072 (*Arachis hypogaea*, ACZ06072); Pvul|ADO17551 (*Phaseolus vulgaris*, ADO17551); Vfor|AEE69020 (*Vernicia fordii*, AEE69020); Vlab|AEI60128 (*Vitis labrusca*, AEI60128); Cole|AFK31315 (*Camellia oleifera*, AFK31315); Cche|AGH32914 (*Camellia chekiangoleosa*, AGH32914). After the sequences were downloaded, the amino acid sequences of the 21 *FAD2* genes together with the deduced peptide sequence of ColeFAD2 gene were aligned using MUSCLE [56] (version 3.8.31) with default parameters. Phylogenetic and molecular evolutionary analyses were constructed based on the alignment using MEGA [43] (version 5.05) with the neighbor-joining (NJ) method. Bootstrap values were calculated from 1,000 iterations. Trees were visualized and modified using EvolView [57].

### Validation by qRT-PCR

Seventeen unigenes involved in lipid metabolism were selected for the validation using real time qPCR. The gene-specific primer pairs were designed using Primer premier 5.0 software (Premier Biosoft International), and all primer sequences were listed in Table S8. Total RNA was isolated from tender shoots and young leaves of *C. oleifera* using a modified CTAB method. cDNA was synthesized using the SuperScript VILO cDNA Synthesis Kit (Invitrogen), according to the manufacturer's guidelines. One microgram of RNA was used in each synthesis reaction. Relative mRNA abundance of the selected unigenes was measured using Multicolor Real-Time PCR Detection System (Bio-Rad). The conditions for all reactions were 95°C for 30 s, 40 cycles of 95°C for 15 s, followed by 60°C for 30 s, and 72°C for 20 s. Melting curve analysis was performed by the end of each PCR to confirm the PCR specificity. Three biological replicates of each reaction were performed, and the translation elongation factor 1-alpha (TEF) gene was chosen as an internal standard for normalization. The quantification of qPCR results for each unigene of interest were calculated using the delta-delta Ct (2^−ΔΔCt^) method. All data were expressed as the mean ± SD after normalization.

## Supporting Information

Figure S1 **Predicted amino acid sequence of ColeFAD2 (ColeIsotig4522: 451–1599) and alignment with Cole|AFK31315 (** ***C. oleifera*** **, AFK31315) and Cche|AGH32914 (** ***C. chekiangoleosa*** **, AGH32914)** ***FAD2*** **genes.** The three point mutations (amino acid: 165, 183 and 348) are show in white background.
(PDF)

Table S1 **Top BLAST hits from NR, UniRef90, TAIR10 and KOG protein databases.** BLAST results for all the transcripts with E-value≤10^−5^ are shown.
(XLS)

Table S2 **Summary of PFAM domains/families in the** ***C. oleifera*** **transcriptome.** PFAM search results for all the unigenes with E-value≤10^−5^ are shown.
(XLS)

Table S3 **Gene Ontology (GO) annotation of the** ***C. oleifera*** **transcripts.** Unigenes with the best matches from NR database were further assigned with GO terms using Blast2GO package.
(XLS)

Table S4 **List of SSR motifs detected in the assembled isotigs of** ***C. oleifera*** **.** Five types of SSRs were identified from the *C. oleifera* transcriptome using the MISA Perl script. This list contains the name of unigene, length, SSR number, motif (SSR type), SSR, SSR size, SSR start and SSR end.
(XLS)

Table S5 **Overview of the SNPs detected in the assembled isotigs of** ***C. oleifera*** **.** This file contains the detailed information for 20,250 putative SNPs identified from *C. oleifera* isotigs.
(XLS)

Table S6 **Summary of the 211 orthologous gene pairs (** ***Ka/Ks*** **ratio >1) between** ***C. oleifera*** **and** ***C. sinensis*** **.** The sequence similarity, nonsynonymous substitution rate (*Ka*), synonymous substitution rate (*Ks*), omega value (*Ka*/*Ks*), and NR annotations are shown.
(XLS)

Table S7 **Detailed information of the lipid metabolism-related genes identified in the** ***C. oleifera*** **transcriptome.** The pathway information, family name, family abbreviation, EC number, isoform/gene specific name, isoform/gene specific abbreviation, subcellular location, TAIR10 gene locus, number of unigenes, average unigene length and the corresponding unigene IDs are given.
(XLS)

Table S8 **Primer pairs of the candidate lipid-related unigenes designed for qRT-PCR.** Specific primer pairs of seventeen candidate unigenes with potential roles in lipid metabolism designed for real time qRT-PCR using the Primer premier software (version 5.0) are shown.
(XLS)
