# Supplementary material for: Transcriptome Analysis of the Oil-Rich Tea Plant, Camellia oleifera, Reveals Candidate Genes Related to Lipid Metabolism
Source: PLoS One. 2014 Aug 19;9(8):e104150. doi: 10.1371/journal.pone.0104150 (PMC4138098; doi:10.1371/journal.pone.0104150)
Supplement: Figure S1 — Predicted amino acid sequence of ColeFAD2 (ColeIsotig4522: 451–1599) and alignment with Cole|AFK31315 ( C. oleifera , AFK31315) and Cche|AGH32914 ( C. chekiangoleosa , AGH32914) FAD2 genes. The three point mutations (amino acid: 165, 183 and 348) are show in white background. (PDF) [file pone.0104150.s001.pdf]

```
                                     10        20        30
                             ....|....|....|....|....|....|
ColeIsotig4522|ColeFAD2      MGAGGRMSVPPEGKKSDRDVIRRVPYSKPP
Cche|AGH32914                MGAGGRMSVPPEGKKSDRDVIRRVPYSKPP
Cole|AFK31315                MGAGGRMSVPPEGKKSDRDVIRRVPYSKPP

                                     40        50        60
                             ....|....|....|....|....|....|
ColeIsotig4522|ColeFAD2      FTVGEIKKAIPPHCFRRSVLHSFSYVVYDL
Cche|AGH32914                FTVGEIKKAIPPHCFRRSVLHSFSYVVYDL
Cole|AFK31315                FTVGEIKKAIPPHCFRRSVLHSFSYVVYDL

                                     70        80        90
                             ....|....|....|....|....|....|
ColeIsotig4522|ColeFAD2      IIAFLFYYLATNYIHLLPQPLSYLAWLVYW
Cche|AGH32914                IIAFLFYYLATNYIHLLPQPLSYLAWLVYW
Cole|AFK31315                IIAFLFYYLATNYIHLLPQPLSYLAWLVYW

                                    100       110       120
                             ....|....|....|....|....|....|
ColeIsotig4522|ColeFAD2      ICQGCVLTGVWVVAHECGHHAFSDYQWLDD
Cche|AGH32914                ICQGCVLTGVWVVAHECGHHAFSDYQWLDD
Cole|AFK31315                ICQGCVLTGVWVVAHECGHHAFSDYQWLDD

                                    130       140       150
                             ....|....|....|....|....|....|
ColeIsotig4522|ColeFAD2      TVGLVLHSALLVPYFSWKYSHRRHHSNTAS
Cche|AGH32914                TVGLVLHSALLVPYFSWKYSHRRHHSNTAS
Cole|AFK31315                TVGLVLHSALLVPYFSWKYSHRRHHSNTAS

                                    160       170       180
                             ....|....|....|....|....|....|
ColeIsotig4522|ColeFAD2      LERDEVFVPKLKSSIGWYSKYLNNPPGRIL
Cche|AGH32914                LERDEVFVPKLKSSIGWYSKYLNNPPGRIL
Cole|AFK31315                LERDEVFVPKLKSSVGWYSKYLNNPPGRIL

                                    190       200       210
                             ....|....|....|....|....|....|
ColeIsotig4522|ColeFAD2      TVVITLTLGWPLYLMFNVSGRHYDRFACHY
Cche|AGH32914                TVIITLTLGWPLYLMFNVSGRHYDRFACHY
Cole|AFK31315                TVVITLTLGWPLYLMFNVSGRHYDRFACHY

                                    220       230       240
                             ....|....|....|....|....|....|
ColeIsotig4522|ColeFAD2      DPYGPIYSDRERLQIYLSDAGVLGVSYVLY
Cche|AGH32914                DPYGPIYSDRERLQIYLSDAGVLGVSYVLY
Cole|AFK31315                DPYGPIYSDRERLQIYLSDAGVLGVSYVLY

                                    250       260       270
                             ....|....|....|....|....|....|
```

```
ColeIsotig4522|ColeFAD2   RFALVKGLAWVLCLYGGPLLIVNGFLVLIT
Cche|AGH32914             RFALVKGLAWVLCLYGGPLLIVNGFLVLIT
Cole|AFK31315             RFALVKGLAWVLCLYGGPLLIVNGFLVLIT

                                   280       290       300
                          ....|....|....|....|....|....|
ColeIsotig4522|ColeFAD2   WLQHTHPALPHYDSSEWDWLRGALATCDRD
Cche|AGH32914             WLQHTHPALPHYDSSEWDWLRGALATCDRD
Cole|AFK31315             WLQHTHPALPHYDSSEWDWLRGALATCDRD

                                   310       320       330
                          ....|....|....|....|....|....|
ColeIsotig4522|ColeFAD2   YGILNKVFHNITDTHVAHHLFSTMPHYHAM
Cche|AGH32914             YGILNKVFHNITDTHVAHHLFSTMPHYHAM
Cole|AFK31315             YGILNKVFHNITDTHVAHHLFSTMPHYHAM

                                   340       350       360
                          ....|....|....|....|....|....|
ColeIsotig4522|ColeFAD2   EATKAIKPILGDYYQCDGTPVLRAIWREAK
Cche|AGH32914             EATKAIKPILGDYYQCDGTPVLRAIWREAK
Cole|AFK31315             EATKAIKPILGDYYQCDATPVLRAIWREAK

                                   370       380
                          ....|....|....|....|...
ColeIsotig4522|ColeFAD2   ECIYVENDESDQTKGVFWYKNKL
Cche|AGH32914             ECIYVENDESDQTKGVFWYKNKL
Cole|AFK31315             ECIYVENDESDQTKGVFWYKNKL
```
